# Neue S3-Leitlinie Prostatakarzinom 2021 (Version 6.2) – Was hat sich beim fortgeschrittenen Prostatakarzinom geändert?

**DOI:** 10.1007/s00120-022-01927-z

**Published:** 2022-09-06

**Authors:** C. Thomas, A. J. Schrader

**Affiliations:** 1 Klinik für Urologie, Universitätsklinikum Carl Gustav Carus, Fetscherstr. 74, 01307 Dresden, Deutschland; 2 Klinik für Urologie und Kinderurologie, Universitätsklinikum Münster, Münster, Deutschland

**Keywords:** Prostatakarzinom, Systemtherapie, Lokaltherapie, Hormonsensitiv, Kastrationsresistent, Prostate cancer, Systemic therapy, Local therapy, Hormone-sensitive, Castration-resistant

## Abstract

Zur Therapie des metastasierten hormonsensitiven oder kastrationsresistenten Prostatakarzinoms (mHSPC, mCRPC) gab es in den letzten Jahren zahlreiche neue Erkenntnisse aus klinischen Studien. Die neu zugelassenen Behandlungsoptionen machen die Therapieplanung und Therapiesequenz anspruchsvoller. Hinzu kommt, dass die Lokaltherapie des metastasierten Prostatakarzinoms zunehmend an Bedeutung gewinnt. Im neuen Update 6.2 der S3-Leitlinie zum Prostatakarzinom vom Oktober 2021 wurden die neuen Entwicklungen bei den Empfehlungen zur Behandlung des mHSPC und mCRPC umgesetzt, deren wichtigsten Neuerungen und die daraus resultierenden Empfehlungen für die Praxis dargestellt werden.

## Abstract (en)

There have been numerous new findings from clinical trials in recent years regarding the treatment of metastatic hormone-sensitive or castration-resistant prostate cancer. The newly approved treatment options make therapy planning and therapy sequencing more challenging. In addition, local therapy of metastatic prostate cancer is becoming increasingly important. In the new German guidelines on prostate cancer (version 6.2, October 2021), new developments in the recommendations for the treatment of mHSPC and mCRPC were implemented, and their most important resulting recommendations for the clinical practice are presented in this review.

## Einführung

Im Oktober 2021 wurde die aktualisierte 6. Fassung der S3-Leitlinie Prostatakarzinom veröffentlicht. Sie dient als Behandlungsleitfaden für die mit jährlich rund 60.000 Neuerkrankungen in Deutschland häufigste Krebserkrankung bei Männern. Als sog. „living guideline“ werden die Empfehlungen regelmäßig auf Aktualität überprüft und bei Bedarf aktualisiert. In diesem Review werden die wichtigsten Änderungen und Neuerungen in den Bereichen systemische Therapie des metastasierten hormonsensitiven und kastrationsresistenten Prostatakarzinoms (mHSPC, mCRPC) sowie Lokaltherapien des oligometastasierten Prostatakarzinoms und die daraus resultierenden neuen Leitlinienempfehlungen für die Praxis dargestellt. Diese betreffen u. a. die Zulassungen von Apalutamid und Enzalutamid, jeweils in Kombination mit einer Androgendeprivationstherapie (ADT), als Ergänzung zu den bereits bestehenden und aktualisierten Empfehlungen der Kombination ADT plus Abirateronacetat/Prednison oder Prednisolon (Abirateron/Prednison) bzw. plus Docetaxel beim mHSPC.

## Systemische Therapie des mHSPC

Gemäß der aktuellen S3-Leitlinie stellt für Patienten mit mHSPC im guten Allgemeinzustand (ECOG 0-1) die Kombinationstherapie der ADT mit den neuen hormonellen Substanzen Abirateron/Prednison, Apalutamid oder Enzalutamid bzw. mit Docetaxel heutzutage die Therapie der Wahl dar [1]. Die ADT-Monotherapie hat keinen Stellenwert mehr, außer bei ausgewählten Patienten, die sich z. B. aufgrund von Komorbiditäten nicht für eine Kombinationstherapie eignen oder diese ablehnen [1].

Alle vier Kombinationstherapien führten in den verschiedenen zulassungsrelevanten Phase-III-Studien (LATITUDE, TITAN, ARCHES, CHAARTED) zu einer ähnlichen Verlängerung des Gesamtüberlebens (OS) im Vergleich zu alleiniger ADT [2–5]. In der LATITUDE-Studie lag das mediane OS in der finalen Analyse (medianer Follow-up 51,8 Monate) unter Abirateron/Prednison plus ADT bei 53,3 vs. 36,5 Monaten unter ADT (Hazard Ratio [HR]: 0,66; *p* < 0,0001; [2]). In der TITAN-Studie war bereits in der ersten Zwischenanalyse (medianer Follow-up 22,7 Monate) ein signifikanter OS-Vorteil mit Apalutamid plus ADT gegenüber ADT festzustellen (HR: 0,67; *p* = 0,005; 2‑Jahres-OS: 82,4 % vs. 73,5 %; medianes OS in beiden Armen noch nicht erreicht; [3]). In der finalen OS-Analyse nach einem medianen Follow-up von 44 Monaten war das Risiko zu sterben trotz Crossover im Apalutamid-Arm um 35 % reduziert (HR: 0,65; *p* < 0,0001; medianes OS noch nicht erreicht vs. 52,2 Monate; [4]). Auch in der finalen Analyse der ARCHES-Studie war nach einem medianen Follow-up von 44,6 Monaten das OS unter Enzalutamid plus ADT signifikant länger als unter alleiniger ADT, wobei das OS im Gegensatz zu den übrigen Studien ein sekundärer Studienendpunkt war (HR: 0,66; *p* = 0,0001; medianes OS in beiden Armen noch nicht erreicht; [5]). In der CHAARTED-Studie betrug das mediane OS nach median 53,7 Monaten unter Docetaxel plus ADT 51,2 vs. 34,4 Monate unter alleiniger ADT (HR: 0,63; *p* < 0,001; [6]). Direkt vergleichende Studien zwischen Abirateron/Prednison, Apalutamid, Enzalutamid und Docetaxel existieren bisher nicht. In Abhängigkeit von Patientenwunsch, Nebenwirkungsspektrum, Komorbiditäten des Patienten, nachfolgenden potenziellen Therapieoptionen sowie zugelassener Indikation kann daher zwischen den verschiedenen Kombinationen individuell gewählt werden.

## Unterschiede in der zugelassenen Indikation und beim Empfehlungsgrad

Hinsichtlich der Anwendung in der klinischen Praxis ist zu beachten, dass Abirateron/Prednison (plus ADT) entsprechend der in der LATITUDE-Studie eingeschlossenen Patientenpopulation nur für das neu diagnostizierte (de novo) High-risk-mHSPC zugelassen ist [7] und in der S3-Leitlinie für diese Indikation eine „Soll“-Empfehlung vorliegt [1]. „High risk“ bedeutet, dass mindestens zwei der drei LATITUDE-Kriterien – Gleason-Score 8–10 oder mindestens drei Knochenmetastasen oder viszerale Metastasen – vorliegen. Für Docetaxel besteht in der EU seit Oktober 2019 die Zulassungserweiterung für die mHSPC-Erstlinientherapie unabhängig von der Metastasenlast („high“ und „low volume“; [8]). Da sich aber in der CHAARTED-Studie ein deutlicher Vorteil von Docetaxel (plus ADT) im Vergleich zur alleinigen ADT v. a. bei High-volume-Patienten zeigte, besteht für Docetaxel in der aktuellen S3-Leitlinie beim High-volume-mHSPC eine „Soll“-Empfehlung und beim Low-volume-mHSPC aufgrund der Evidenzlage sowie dem im Vergleich zu den neuen Hormonsubstanzen schlechteren Toxizitätsprofil nur eine „Kann“-Empfehlung, wobei die Patienten über die im Vergleich zu den neuen Hormonsubstanzen höhere Toxizität aufgeklärt werden sollen [1]. „High volume“ war definiert als mindestens vier Knochenmetastasen, davon mindestens eine außerhalb des Achsenskeletts bzw. Beckens und/oder viszerale Metastasen [3]. Enzalutamid und Apalutamid sind entsprechend der in den Zulassungsstudien eingeschlossenen Patientenpopulationen bei einem breiten Spektrum von mHSPC-Patienten unabhängig von Metastasenlast oder Risikostatus zugelassen [9, 10] und haben in der Leitlinie eine „Soll“-Empfehlung [1]. Demzufolge empfiehlt die aktuelle S3-Leitlinie die Patienten auch weiterhin nach „high/low risk“ (Definition nach LATITUDE) und „high/low volume“ (Definition nach CHAARTED) zu stratifizieren [1].

In der Patientenaufklärung sollten die einzelnen Therapieoptionen ausführlich erläutert werden, insbesondere auch hinsichtlich der im Vergleich zu den neuen Hormonsubstanzen höheren Toxizität der Chemotherapie mit Docetaxel [1]. Dies scheint sich auch in der Lebensqualität widerzuspiegeln: Die anfänglich durchgehend hohe Lebensqualität der mHSPC-Patienten wurde durch die zusätzliche Gabe von Abirateron/Prednison, Apalutamid oder Enzalutamid nicht beeinträchtigt [4, 11, 12]. Bei Docetaxel zeigte sich in der CHAARTED-Studie, dass die assoziierten Nebenwirkungen mit einer vorübergehenden Verschlechterung der Lebensqualität während der Chemotherapie einhergehen [13].

## Therapie des oligometastasierten mHSPC

Das oligometastasierte Prostatakarzinom stellt eine Sonderform im metastasierten Stadium dar, definiert durch das Vorhandensein von nur wenigen Knochenmetastasen und noch keinen Organmetastasen in der konventionellen Bildgebung (Skelettszintigraphie und CT oder MRT). Sie gilt auch als intermediäres Stadium zwischen lokal begrenzter und systemisch ausgedehnter Metastasierung. Bislang existiert keine einheitliche Definition für dieses Stadium der Frühmetastasierung. Zumeist ist aber je nach Studie eine limitierte Metastasierung von maximal 3 bis maximal 5 ossären und/oder lymphogenen Fernmetastasen gemeint. Die S3-Leitlinie hat auf Basis der aktuellen Evidenz das Stadium der Oligometastasierung in einem neuen Kapitel zur lokalen Therapie bei metastasiertem Prostatakarzinom berücksichtigt und eine Definition des oligometastasierten Prostatakarzinoms eingeführt. Diese beschränkt die Anzahl der Metastasen in der konventionellen Bildgebung auf maximal 4 Knochenmetastasen. Extraossäre viszerale Metastasen dürfen nicht vorliegen. Als viszerale Metastasen gelten Weichteilmetastasen in Leber, Lunge, Gehirn und anderen Organen, nicht jedoch lymphogene Fernmetastasen [1].

Zu unterscheiden ist die primäre Oligometastasierung (Metastasen bereits bei Diagnose des Primärtumors vorhanden) von der sekundären Oligometastasierung (Auftreten von Metastasen nach Lokaltherapie und Progress). Die technologischen Fortschritte in der Bildgebung mit verbesserten sensitiven Verfahren wie die PSMA-PET-CT haben in den letzten Jahren dazu geführt, dass nun öfter oligometastasierte Patienten diagnostiziert werden [14–16]. Diese Patienten können von einer metastasengerichteten Therapie („metastasis directed therapy“, MDT) oder einer lokalen Therapie des Primärtumors (perkutane Strahlentherapie, evtl. auch radikale Prostatektomie, RPE) profitieren. Laut S3-Leitlinie sollten Patienten mit primär oligometastasiertem Prostatakarzinom zusätzlich zur systemischen Therapie eine hypofraktionierte perkutane Strahlentherapie der Prostata erhalten [1]. Für die RPE ist die Evidenzlage noch unzureichend, kann aber nach Diskussion im Tumorboard im Rahmen einer multimodalen Therapie erwogen werden. Eine MDT kann eine Progression und damit den Beginn einer palliativen Systemtherapie hinauszögern und sollte gemäß Leitlinie vorzugsweise als stereotaktische ablative Radiotherapie (SABR) erfolgen [1].

### Metastasengerichtete lokal ablative Therapie

Für das Prostatakarzinom liegen aktuell 2 randomisierte Phase-II-Studien zum Nutzen einer MDT für sekundär oligometastasierte Patienten vor. In der STOMP-Studie mit 62 asymptomatischen mHSPC-Patienten nach Lokaltherapie im PSA-Progress mit maximal 3 Metastasen im Cholin-PET-CT war die Zeit bis zur Einleitung einer ADT (primärer Endpunkt) in der Gruppe mit MDT (SABR oder Resektion) deutlich länger bei ähnlicher Lebensqualität als in der Gruppe, die lediglich beobachtet wurden (medianes ADT-freies Überleben: 21 vs. 13 Monate; HR: 0,60; *p* = 0,11; [17]). Die 5‑Jahres-Rate für ADT-freies Überleben betrug 34 % in der MDT-Gruppe gegenüber 8 % in der Kontrollgruppe (HR: 0,57; *p* = 0,06; [18]). Die Einleitung einer ADT erfolgte bei symptomatischer Progression, bei Auftreten neuer Metastasen oder bei Progression bestehender Metastasen.

Die kürzlich publizierten Ergebnisse der ORIOLE-Studie, in der 54 asymptomatische mHSPC-Patienten nach Lokaltherapie im PSA-Progress mit ebenfalls maximal 3 Metastasen jedoch in der konventionellen Bildgebung eingeschlossen waren, zeigten einen signifikanten Unterschied im primären Endpunkt Progression nach 6 Monaten zugunsten der SABR (19 % vs. 61 % bei Beobachtung; *p* = 0,005; [19]). Progression war definiert als PSA-Anstieg, Progression in der konventionellen Bildgebung, symptomatische Progression, Einleitung einer ADT oder Tod. Das mediane PFS war im SABR-Arm noch nicht erreicht und lag im Kontrollarm bei 5,8 Monaten (*p* = 0,002).

### Lokale Therapie des Primärtumors

Bei Patienten mit einem neu diagnostizierten (primär) oligometastasierten Prostatakarzinom stellt sich immer wieder die Frage, ob zusätzlich zu einer palliativen Systemtherapie eine lokale Therapie der Prostata mittels perkutaner Strahlentherapie oder radikaler Prostatektomie (RPE) erfolgen sollte. Während der Nutzen für eine perkutane Strahlentherapie des Primärtumors bei Oligometastasierung gezeigt werden konnte und gemäß der aktuellen Leitlinie bei entsprechenden Patienten zusätzlich zur systemischen Therapie durchgeführt werden sollte [1], ist der Stellenwert der RPE noch ungeklärt. Mehrere retrospektive Studien weisen bereits auf einen möglichen Überlebensvorteil der RPE beim oligometastasierten Prostatakarzinom hin [20–24]. Es liegen aber bisher noch keine abgeschlossenen randomisierten Studien vor. Erste Zwischenergebnisse einer laufenden randomisierten Phase-II-Studie aus Asien, die bei 200 primär oligometastasierten mHSPC-Patienten mit maximal 5 Knochenmetastasen (96 %) oder Lymphknotenmetastasen (15 %) eine ADT plus Lokaltherapie des Primärtumors (*n* = 100; davon 85 mit RPE und 11 mit Strahlentherapie) gegenüber ADT vergleicht, zeigten nach einem medianen Follow-up von 28 Monaten einen radiologischen Progress bei 19 % der Patienten unter ADT plus Lokaltherapie vs. 33 % unter alleiniger ADT [25]. Das mediane radiologische PFS (primärer Endpunkt) lag unter ADT bei 50 Monaten und war unter ADT plus Lokaltherapie noch nicht erreicht (HR: 0,50; *p* = 0,013). Für eine Auswertung des Gesamtüberlebens (sekundärer Endpunkt) war es noch zu früh. Hingewiesen sei noch darauf, dass eine Kombination der ADT mit Bicalutamid oder Flutamid möglich war, nicht jedoch eine Kombination mit einer neuen hormonellen Substanz (Abirateron/Prednison, Apalutamid oder Enzalutamid). Aufgrund der unzureichenden Evidenz hat die RPE in der neuen S3-Leitlinie zwar eine „Kann“-Empfehlung, jedoch unter dem Vorbehalt einer Beratung in einem interdisziplinären Tumorboard [1]. Erwogen wird eine RPE beim oligometastasierten Prostatakarzinom derzeit v. a., um lokalen progressionsbedingten Komplikationen im Bereich des unteren Harntraktes vorzubeugen [1, 20, 21].

## Nachfolgetherapien bei Fortschreiten zum mCRPC

Für Patienten mit asymptomatischem oder gering symptomatischem mCRPC und Progression unter ADT sind aktuell Abirateron/Prednison, Enzalutamid oder Docetaxel zugelassen und werden auch von der S3-Leitlinie empfohlen, allerdings mit einem unterschiedlichen Empfehlungsgrad [1]. Während die Leitlinie für Abirateron/Prednison und Enzalutamid eine „Soll“-Empfehlung ausspricht, hat Docetaxel nur die schwächere „Kann“-Empfehlung, weil in den zulassungsrelevanten Studien mit Docetaxel überwiegend symptomatische Patienten eingeschlossen waren. Bei Progress unter einer neuen hormonellen Substanz sollte ein Wechsel der Therapiestrategie erfolgen [1]. Nach Docetaxel kann bei Patienten mit gutem Allgemeinzustand Abirateron/Prednison, Enzalutamid oder Cabazitaxel, bei Bedarf in Kombination mit symptombezogener und supportiver Therapie, zum Einsatz kommen [1].

Mit dem PARP-Inhibitor Olaparib steht seit November 2020 erstmals auch beim Prostatakarzinom eine molekular gezielte Therapie zur Verfügung. Zugelassen ist Olaparib für Patienten mit mCRPC und DNA-Reparaturdefekten (*BRCA1/2*-Mutation) nach antihormoneller Vorbehandlung mit einer der neuen Hormonsubstanzen [26]. Entsprechend sollen gemäß Leitlinie alle mCRPC-Patienten nach einer Vortherapie mit einer neuen hormonellen Substanz auf *BCRA1/2*-Mutationen getestet werden, so dass im Falle eines entsprechenden Mutationsnachweises die Möglichkeit der Therapie mit Olaparib besteht [1]. Olaparib verlängerte in der Phase-III-Zulassungsstudie PROfound in der Subgruppe der 160 mCRPC-Patienten mit *BRCA1/2*-Mutation, die unter einer neuen hormonellen Substanz wie Abirateron oder Enzalutamid nach Wahl des Prüfarztes eine Progression erlitten, signifikant das mediane rPFS (7,4 vs. 3,6 Monate; HR: 0,34; *p* < 0,001) und das mediane OS (19,1 vs. 14,7 Monate; HR: 0,69; *p* = 0,02) verglichen mit einer erneuten Gabe von Abirateron/Prednison oder Enzalutamid [27].

Die langetablierten mCRPC-Studien, die nach Versagen einer einfachen ADT erhoben wurden, bilden nur noch bedingt den aktuellen Therapiestandard ab. Da die mHSPC-Therapie immer häufiger als Kombinationstherapie durchgeführt wird, richtet sich die mCRPC-Therapie Prä-Chemo und die nachfolgenden Therapien u. a. nach der Vortherapie des mHSPC. Bisher existieren dazu aber noch keine prospektiv-randomisierten Studien und nur wenige retrospektive Untersuchungen. Erfolgte die mHSPC-Therapie mit Docetaxel plus ADT wird nach Progress zum mCRPC die Gabe von Abirateron/Prednison oder Enzalutamid empfohlen [1]. Eine Docetaxel-Reexposition wird hingegen nicht empfohlen, weil sich in einer retrospektiven Analyse der Phase-III-Studie GETUG-AFU 15 ein starker Wirkverlust zeigte (PSA50-Ansprechen: 20 % vs. 53 % mit Abirateron/Prednison oder Enzalutamid; [28]). In einer anderen retrospektiven Analyse zeigte sich nach Docetaxel plus ADT im mHSPC unter Abirateron/Prednison oder Enzalutamid in der mCRPC-Erstlinie ein medianes rPFS von 9 Monaten gegenüber 3 Monaten unter nichthormoneller Therapie [29].

Nach Apalutamid plus ADT im mHSPC lieferte eine vorab geplante explorative PFS2-Analyse der TITAN-Studie Hinweise darauf, dass die Wirksamkeit einer nachfolgenden mCRPC-Therapie erhalten bleibt. So zeigte sich bereits in der Interimsanalyse im Apalutamid-Arm ein signifikant längeres PFS2 als im Placeboarm (HR: 0,66; *p* = 0,0026; [3]). In einer Post-hoc-Analyse konnte ein signifikant längeres PFS2 sowohl für die Folgetherapie mit Abirateron/Prednison (HR: 0,684; *p* = 0,0326) als auch mit Docetaxel (HR: 0,634; *p* = 0,0062) gezeigt werden [30]. Zur mCRPC-Therapie nach Abirateron/Prednison plus ADT im mHSPC liegen bisher keine Studien vor.

## Fazit für die Praxis

- Derzeit sind Abirateron/Prednison, Enzalutamid, Apalutamid und Docetaxel jeweils als Kombination mit ADT zur Therapie des mHSPC zugelassen.
- Beim oligometastasierten mHSPC ist eine systemische Tumortherapie Grundpfeiler der multimodalen Therapie.
- Oligometastasierte Patienten profitieren genauso von der Addition neuartiger hormoneller Substanzen wie Patienten mit einer hohen Metastasenlast.
- Eine Lokaltherapie der Prostata wird bei Oligometastasierung (maximal 3 Knochenmetastasen in der konventionellen Bildgebung) empfohlen. In Einzelfällen kann eine zusätzliche metastasengerichtete Therapie von Vorteil sein.
- Es existieren keine prospektiv-randomisierten Studien nach Versagen einer modernen Kombinationstherapie im mHSPC-Stadium.
- Nach Versagen von Docetaxel plus ADT im mHSPC wird aktuell die Gabe von Abirateron/Prednison oder Enzalutamid empfohlen.
- Nach Versagen einer Apalutamid-Therapie im mHSPC scheinen sowohl Taxane als auch neue antihormonelle Substanzen wirksam zu sein.
- Olaparib gilt als neue Therapieoption beim mCRPC mit nachgewiesenem Reparaturgendefekt (BRCA1/2-Mutation) nach dem Einsatz mindestens einer neuen hormonellen Substanz.
